# Nuclear COMMD1 Is Associated with Cisplatin Sensitivity in Ovarian Cancer

**DOI:** 10.1371/journal.pone.0165385

**Published:** 2016-10-27

**Authors:** Alina Fedoseienko, Hylke W. Wieringa, G. Bea A. Wisman, Evelien Duiker, Anna K. L. Reyners, Marten H. Hofker, Ate G. J. van der Zee, Bart van de Sluis, Marcel A. T. M. van Vugt

**Affiliations:** 1 Department of Pediatrics, Molecular Genetics Section, University of Groningen, University Medical Center Groningen, Groningen, The Netherlands; 2 Department of Medical Oncology, University of Groningen, University Medical Center Groningen, Groningen, The Netherlands; 3 Department of Pathology, University of Groningen, University Medical Center Groningen, Groningen, The Netherlands; 4 Department of Gynecological Oncology, University of Groningen, University Medical Center Groningen, Groningen, The Netherlands; Universite du Quebec a Trois-Rivieres, CANADA

## Abstract

Copper metabolism MURR1 domain 1 (COMMD1) protein is a multifunctional protein, and its expression has been correlated with patients’ survival in different types of cancer. *In vitro* studies revealed that COMMD1 plays a role in sensitizing cancer cell lines to cisplatin, however, the mechanism and its role in platinum sensitivity in cancer has yet to be established. We evaluated the role of COMMD1 in cisplatin sensitivity in A2780 ovarian cancer cells and the relation between COMMD1 expression and response to platinum-based therapy in advanced stage high-grade serous ovarian cancer (HGSOC) patients. We found that elevation of nuclear COMMD1 expression sensitized A2780 ovarian cancer cells to cisplatin-mediated cytotoxicity. This was accompanied by a more effective G_2_/M checkpoint, and decreased protein expression of the DNA repair gene *BRCA1*, and the apoptosis inhibitor *BCL2*. Furthermore, COMMD1 expression was immunohistochemically analyzed in two tissue micro-arrays (TMAs), representing a historical cohort and a randomized clinical trial-based cohort of advanced stage HGSOC tumor specimens. Expression of COMMD1 was observed in all ovarian cancer samples, however, specifically nuclear expression of COMMD1 was only observed in a subset of ovarian cancers. In our historical cohort, nuclear COMMD1 expression was associated with an improved response to chemotherapy (OR = 0.167; *P* = 0.038), although this association could not be confirmed in the second cohort, likely due to sample size. Taken together, these results suggest that nuclear expression of COMMD1 sensitize ovarian cancer to cisplatin, possibly by modulating the G_2_/M checkpoint and through controlling expression of genes involved in DNA repair and apoptosis.

## Introduction

Copper metabolism MURR1 domain 1 (COMMD1) protein is a small ubiquitously expressed protein, which has been shown to effect tumor cell behavior and survival [1–4]. According to the Oncomine database, COMMD1 is differentially expressed in multiple cancer types [5], and decreased *COMMD1* expression in endometrial cancer tissue was shown to correlate with a worse overall survival [1]. One of the proposed mechanisms of COMMD1 in mediating the behavior of cancer involves its inhibitory action on the activity of the transcription factors hypoxia inducible factor 1 (HIF-1) and nuclear factor kappa-light-chain-enhancer of activated B cells (NF-κB) [1,4]. HIF-1 and NF-κB both have a significant role in tumor behavior and clinical outcome, including ovarian cancer [6–8].

Expression of COMMD1 is predominantly observed in the cytoplasm of most cell types, although an important role for COMMD1 in the nucleus has been revealed [9–11]. In complex with an E3 ubiquitin ligase, nuclear COMMD1 promotes the proteasomal degradation of the NF-κB subunit p65, which leads to inhibition of transcriptional activity of NF-κB [12–18]. COMMD1 also represses HIF-1 activity [19,20], and elevated nuclear COMMD1 expression augments its inhibitory effect on both NF-Ф06BB and HIF-mediated transcription [10]. Additionally, nuclear COMMD1 was suggested to participate in the cellular response to DNA damage, through its ability to interact with the DNA repair proteins Breast Cancer 1 Early Onset (BRCA1), BRCA1-associated RING domain protein 1 (BARD1) and Checkpoint kinase 2 (Chk2) [3]. Furthermore, ablation of COMMD1 resulted in increased sensitivity to DNA-damaging agents, including cisplatin, in several cell lines [3,21]. The exact mechanism underlying this effect, and whether COMMD1 expression levels are associated with the response to platinum-based therapy in cancer patients remains unclear.

In this study, we investigated the role of COMMD1 in cisplatin sensitivity, and assessed COMMD1 expression in two cohorts of patients treated for advanced stage ovarian cancer. We show that increased levels of nuclear COMMD1 affect cisplatin sensitivity in ovarian cancer cells *in vitro*, and that nuclear COMMD1 expression in ovarian cancer tumor tissue is associated with improved response to cisplatin treatment in one out of two analyzed patient cohorts.

## Materials and Methods

### Cell lines

Human embryonic kidney HEK293T cells (ATCC, #CRL-3216), HeLa cervical cancer cells (ATCC, #CCL-2) and MBA-MB-231 breast cancer cells (ATCC, #HTB-26) were cultured in Dulbecco’s modified Eagle medium GlutaMAX™, supplemented with 10% fetal calf serum (FCS) and 1% penicillin-streptomycin (pen/strep). The human ovarian cancer cell lines A2780 and the cisplatin-resistant derivative A2780/CP70 were a kind gift of Prof. Steven de Jong and were described previously [22]. A2780, A2780/Cp70 and Peo14 (Sigma, 10032311) ovarian cancer cells were cultured in RPMI medium GlutaMAX™, supplemented with 10% FCS and 1% pen/strep. All cell lines were cultured at 5% CO_2_ and 21% O_2_. The HeLa, HEK293T, A2780 and MDA-MB-231 cell lines in which COMMD1 was stably depleted using shRNA were maintained in media supplemented with puromycin (1 μg/ml) as previously described [1,19,20]. A2780 and Peo14 cell lines stably expressing COMMD1-Flag were generated using the retroviral expression vector pBabe-Puro. To produce retrovirus particles, HEK293T cells were transfected with pBabe-Puro or pBabe-Puro-COMMD1-Flag in combination with packaging plasmids pMDLG-pRRE, pH-CMV-G and pRSV-Rev. Virus-containing supernatant culture medium was filtered (0.45 micron, Corning), mixed with polybrene (4 μg/ml) and used for infection for three consecutive 12-hour periods. Twenty-four hours after the third infection, puromycin was added (1 μg/ml) for selection.

### Western blotting

For Western blotting, total cell lysates were obtained using NP40 buffer (0.1% Nonidet P-40 (NP-40), 0.4 M NaCl, 10 mM Tris-HCl (pH 8.0), 1 mM EDTA) supplemented with protease and phosphatase inhibitors (Roche). For the isolation of cytosolic fractions, cells were lyzed in buffer A (10 mM Hepes (pH 7.9), 10 mM KCl, 0.1 mM EDTA, 0.1 mM EGTA, 0.15% NP-40, 1 mM DTT) with protease and phosphatase inhibitors (Roche). Lysates were centrifuged at 12,000 g for 30 seconds at 4°C, and supernatants were taken as cytosolic fraction. The pellets were washed with ice-cold phosphate-buffered saline (PBS) and lyzed in buffer B (20 mM Hepes (pH 7.9), 400 mM NaCl, 1 mM EDTA, 1 mM EGTA, 0.5% NP-40, 1 mM DTT). After sonication (5 seconds at 50 watt), samples were centrifuged at 12,000 g for 15 minutes at 4°C, and supernatants were taken as nuclear fractions. Protein concentration was determined using Bradford assay (Biorad). Thirty micrograms of protein was separated using SDS-PAGE and transferred to Amersham Hybond-P PVDF Transfer Membrane (GE Healthcare; RPN303F). Membranes were blocked in 5% milk in Tris-buffered saline-0.01% Tween20 and incubated with the indicated antibodies. Membranes were visualized using a ChemiDoc XRS+ System (Bio-Rad) using Image Lab software version 5.2.1 (Bio-rad). Images have been cropped for presentation, but full-size images are presented in S4 Fig.

### Immunofluorescence

A2780-empty vector (EV) and A2780-COMMD1 cells were cultured on coverslips for 24 hours, fixed in ice-cold fixative (4% paraformaldehyde and 0.5% glutaraldehyde in PBS) and incubated for 18 minutes at room temperature in the dark, followed by permeabilization with 0.2% Triton X-100 in PBS for 4 minutes. Subsequently, cells were incubated with 10 μg/ml primary antibody directed against COMMD1 (R&D biosystems; MAB7526) in IF buffer (Tris-buffered saline plus human serum cocktail, Sigma; H4522) overnight at 4°C in a humidifier chamber. After three washes in PBS, cells were incubated with Fluorescein (FITC)-conjugated anti-rabbit antibody (1:300; Jackson Immunoresearch Laboratories; 711095152) for 1 hour at room temperature or overnight at 4°C in a humidifier chamber. After three washes in PBS, coverslips were mounted on slides with Vectashield mounting medium with DAPI (Vector Laboratories; H-1200). Images were obtained with a Zeiss Axio Imager2 with a Plan-APOCHROMAT 63x/1.4 Oil objective, using ZEN software (Zeiss).

### Antibodies

We used the following antibodies: mouse monoclonal anti-COMMD1 (clone 3B3; Abcam; ab131597), rabbit polyclonal anti-COMMD1 (Proteintech Group; 11938-1-AP), mouse monoclonal anti-β-Actin (Sigma-Aldrich; A5441), rabbit polyclonal anti-Tubulin (Abcam; ab4047), rabbit polyclonal anti-Lamin A/C (Cell Signaling Technology; #2032), mouse monoclonal anti-Bcl2 (Santa Cruz Biotechnology; sc-509), rabbit monoclonal anti-PARP (Cell Signaling Technology; #9532), rabbit polyclonal anti-cleaved-caspase3 (Cell Signaling Technology; #9661), mouse monoclonal anti-phospho-Ser10-Histone H3 antibody, (Cell Signaling Technology; #9706), mouse monoclonal anti-XIAP (BD Biosciences, # 610716), rabbit polyclonal anti-BRCA1 (Cell Signaling Technology; #9010), mouse monoclonal anti-Hsp90 (AC88) (Enzo; ADI-SPA-830), rabbit monoclonal anti-phospho-Ser139-H2AX (Cell Signaling Technology; #9718), Alexa-488-conjugated polyclonal anti-mouse (Molecular Probes; A-11001), Alexa-647-conjugated polyclonal anti-rabbit (Molecular Probes, A-21244), HRP-conjugated polyclonal goat anti-rabbit IgG (H + L) (Bio-Rad; #170–6515), HRP-conjugated polyclonal goat anti-mouse IgG (H + L) (Bio-Rad; #170–6516).

### Viability assay

To measure cell viability 2,000 cells were plated in 96-well plates (triplicates for each condition). 24 hours after plating, cells were treated either with control medium or medium containing cisplatin (Tocris) at indicated concentrations for 72 hours. After incubation, 20 μl of 5 mg/ml MTT (3-(4,5-dimethylthiazol-2-yl)-2,5-diphenyltetrazolium bromide) was added for 3 hours. Subsequently, culture medium was removed, and cells were incubated in dimethyl sulphoxide (DMSO) for 30 minutes. Absorbance was measured at 520 nm using a Biorad microplate reader. Viability was measured by calculating relative MTT conversion. MTT conversion of cells treated with control medium was used as a reference.

### Flow cytometry

A2780-EV and A2780-COMMD1 cells were harvested after 24 hours of incubation with 2 μM cisplatin and fixed in ice-cold 70% ethanol. Cells were stained with mouse anti-phospho-Ser10-Histone H3 antibody and with rabbit anti-phospho-Ser139-H2AX and subsequently stained with Alexa-488-conjugated anti-mouse and Alexa-647-conjugated anti-rabbit antibody. In addition, counterstaining was performed with propidium iodide/RNAse (Sigma-Aldrich). Cell cycle distribution, phospho-Histone H3 and anti-phospho-H2AX positivity were analyzed on a FACSCalibur (Becton Dickinson Biosciences) equipped with CellQuest software. Per sample, at least 10,000 events were analyzed, and indicated results show averages and standard deviations of three independent experiments.

### Gene expression analysis

A2780-EV and A2780-COMMD1 cells were grown to 70% confluency and were left untreated or were treated with 2mM cisplatin for 24hrs. Cells were harvested in QIAzol Lysis Reagent (Qiagen), and total RNA was isolated by chloroform extraction. Isopropanol-precipitated and ethanol-washed RNA pellets were dissolved in RNase/DNase free water. One microgram of RNA was used to prepare cDNA with the Transcriptor Universal cDNA Master (Roche), according to the protocol provided by the manufacturer. 20 ng cDNA was used for subsequent quantitative real-time PCR (qRT-PCR) analysis using FastStart SYBR Green Master (Roche) and 7900HT Fast Real-Time PCR System (Applied Biosystems). The following PCR program was used: 50°C/2 minutes, 95°C/10 minutes, 40 cycles of 95°C/15 seconds and 60°C/1 minute. Expression data were analyzed using SDS 2.3 software (Applied Biosystems), using the ‘standard curve’ method of calculation. GAPDH expression was used as an internal control. Primer sequences are listed in S1 Table.

### Patients

Two previously described ovarian cancer tissue microarrays (TMA1 and TMA2) were analyzed in this study [23,24]. TMA1 includes retrospectively collected data and was used as an exploratory TMA, whereas TMA2 includes prospectively collected data and was used to validate our observations from TMA1.

Concerning TMA1: between April 1988 and May 2006, 354 patients were treated in the University Medical Center Groningen (UMCG) for ovarian cancer. Tumor tissues of 232 patients were included on TMA1 as described previously [25]. For this study, follow-up data of these patients were updated and new patients (treated between 2002 and 2006) were additionally included. 126 of these patients were diagnosed with advanced stage HGSOC, and chemo-naïve tumor tissue of these patients was used for exploratory immunohistochemical analysis. After primary surgery, patients received platinum-based chemotherapy (cisplatin or carboplatin, with or without a taxane). Tissue collection and storage of clinicopathological and follow up data was only performed upon patients’ approval via informed consent. Clinical data was collected in the UMCG and stored digitally in a central database, which is solely accessible by two dedicated data managers. Statistical analysis was performed with an anonymized dataset extracted from the central database. Protection of patient identity was thereby warranted and according to Dutch law no further Institutional Review Board approval was necessary. Patient staging was done according to the FIGO (International Federation of Gynaecology and Obstetrics) criteria. Tumor grading and classification was determined by a gynecological pathologist. When sufficient tumor tissue and complete follow up information was available, patient data were considered suitable for inclusion for analysis. We selected advanced stage (stage 3 and 4) HGSOC patients (n = 126). To retrospectively evaluate response to chemotherapy, we defined two groups of patients demonstrating extreme therapy responses; a ‘responder’ and a ‘non-responder group’ as previous described [25]. Patients with advanced stage HGSOC, ≥2 cm residual disease after debulking surgery, adjuvant platinum-based chemotherapy and a progression free survival (PFS) of >18 months were considered ‘responders’. When PFS was <6 months, patients were considered ‘non-responders’.

TMA2 comprises tumor tissues of patients who were treated as part of a Dutch multicenter randomized phase II study [26]. The clinical trial of which patient material was included on TMA2 was registered as NTR1491 in the publically accessible Dutch Trial Registry (trialregister.nl), part of the Dutch Cochrane Centre. Details and results of this study were published in [26]. Data were prospectively collected. In this study, patients were treated with carboplatin/docetaxel and randomized to celecoxib (twice daily 400 mg) or a placebo treatment. In short, patients with histopathological conformation of epithelial ovarian cancer, fallopian tube or primary peritoneal carcinomas, FIGO IC-IV, were included. The primary study objectives were response rate and PFS. Response rate was determined using CA125 levels and the Response Evaluation Criteria in Solid Tumors (RECIST version 1.0). Between March 2003 and November 2008, a total of 202 patients enrolled the study, of which 196 were eventually included for statistical analysis. Written informed consent was provided before patients were included. No differences in patient outcome between the two study arms were observed. Sufficient tumor specimens of 121 out of 196 patients were available for immunohistochemical analysis. Of these patients, 67 were diagnosed with advanced stage HGSOC. To test our hypothesis from TMA1, a ‘responder’ and a ‘non-responder’ group was defined in a similar way as for TMA2. However, instead of 2 cm residual disease after debulking surgery, the presence of residual disease was used as a metric for response to treatment.

For the patients obtained from the UMCG in The Netherlands, patients gave informed consent for collection and storage of tissue samples in a tissue bank for future research. All relevant patient data were retrieved and transferred into an anonymous, password-protected, database. The patients' identity was protected by study-specific, unique patient codes and their true identity was only known to two dedicated data managers. According to Dutch regulations, these precautions meant no further institutional review board approval was needed (http://www.federa.org/). TMA1 is not prospectively collected, TMA2 is prospectively collected, but not for the purpose of this study. From the co-authors, Anna K.L. Reyners and Ate G.J. van der Zee are treating physicians, although they did not treat all patients themselves.

### Immunohistochemistry

**Fig 1:**
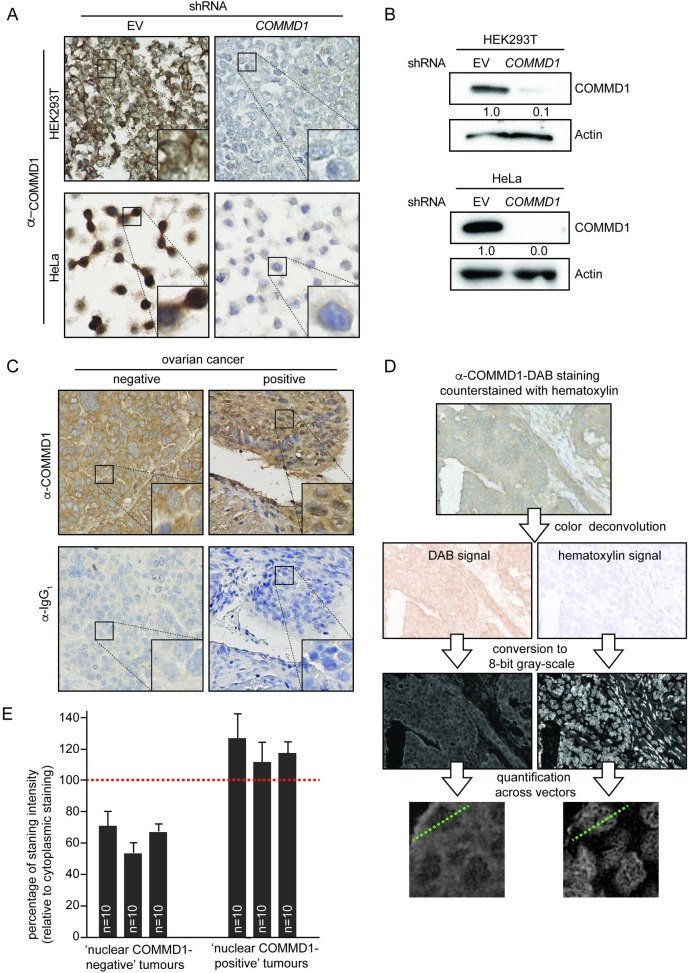
Antibody validation and exploratory immunostaining for COMMD1 in human ovarian tumor samples. **(A)** Representative immunohistochemical COMMD1 staining in paraffin embedded HEK293T and HeLa cells depleted for COMMD1. **(B)** HEK293T and HeLa cells were stably silenced for COMMD1 as shown by immunoblotting. (**C**) Observational immunostainings for COMMD1, including its control IgG_1_ immunostaining in a consecutive slide, in HGSOC patient samples demonstrating either absent or presence of nuclear COMMD1. (**D**) Quantification workflow of immunohistochemical COMMD1 staining. Image analysis was performed using the ImageJ-based software package FIJI. DAB staining and hematoxylin staining were deconvoluted and images were subsequently converted into 8-bit gray scale images. Hematoxylin staining was used to define cytoplasm/nucleus boundaries. Vectors were subsequently used to measure DAB staining intensities across cells and quantify nuclear COMMD1 levels in relation to cytoplasmic levels. (B) Three ‘nuclear COMMD1-negative’ (n = 10 cells per tumor sample) and three ‘nuclear COMMD1-positive’ tumor samples were analyzed. Averages and standard deviations are indicated. Relative nuclear COMMD1 levels to cytoplasmic levels are plotted per tumor sample.

TMA1 and TMA2 were constructed as previously described [23,24,26]. For immunohistochemistry, 4 μm sections were de-paraffinized in xylene. Subsequently, antigen retrieval was performed by 15 minutes boiling in TRIS/EDTA (pH 9.0) buffer. Endogenous peroxidase was blocked by 0.3% hydrogen peroxide for 30 minutes. COMMD1 was stained using mouse monoclonal anti-COMMD1 (1:50; clone 3B3; Abcam, ab131597). Detection was conducted using a horseradish peroxidase (HRP) conjugated secondary rabbit anti-mouse antibody (1:100; DAKO; P0260) and a HRP-conjugated tertiary goat anti-rabbit antibody (1:100; DAKO; P0448). Staining was visualized with 3’3-diaminobenzidine tetrahydrochloride and counterstained with hematoxylin. HEK293T and HeLa cell lines, in which COMMD1 expression was stably depleted using shRNA-mediated COMMD1 knockdown, were used as negative controls for immunohistochemistry [1]. Cells were embedded in paraffin, sliced in 4 μm sections and subsequently mounted on amino-propyl-ethoxy-silan-coated glass slides. Slides were incubated with mouse monoclonal anti-COMMD1 (1:100; clone 3B3; Abcam; ab131597) following the similar protocol as described above. Twenty-one randomly selected tumor samples from ovarian cancer patients were used to stain whole section for exploratory analysis of COMMD1 immunohistochemistry (Fig 1C). Statistical analysis of this group of staining was not performed due to small samples size, and because these tumors are also part of TMA1. Quantification workflow of immunohistochemical COMMD1 staining.

For semi-quantitative analysis, immunohistochemical slides were scanned on a Hamamatsu NanoZoomer, and saved as TIFF files. Image analysis was subsequently performed using the ImageJ-based software package FIJI [27]. 3,3'-Diaminobenzidine (DAB) staining and hematoxylin staining were deconvoluted using ‘colour deconvolution’ with ‘H DAB’ settings. Images were subsequently converted into 8-bit gray scale images and inverted. Values of DAB staining were left unaltered, whereas hematoxylin staining was contrast enhanced to define cytoplasm/nucleus boundaries. Vectors with a length of ~100 pixels were subsequently used to measure DAB staining intensities across cells.

### Evaluation of staining

Cytoplasmic and nuclear COMMD1 expression levels were semi-quantitative scored by two independent observers (AF and HWW) as previously described [28]. Patients were included for statistical analysis if ≥ 2 cores (out of 4) were evaluable for COMMD1 expression. Observers had a concordance in scores in > 90% of the cases. Discordances in scoring were reviewed by AF and HWW to reach consensus for a definitive score. The total immunoreactive score (IRS, 0–8) was constituted by adding the score of staining intensity (0–3) with the percentage of tumor stained (0–5). Staining intensity was scored as negative (0), weak positive (1), positive (2), strong positive (3). Subsequently, percentage of tumor cells stained was scored as 0% (0), 0–5% (1), 5–25% (2), 25–50% (3), 50–75% (4), >75% (5). Scores for cytoplasmic COMMD1 were arbitrary divided into ‘low’ (IRS = 0–6) and ‘high’ (IRS ≥7), while scores for nuclear COMMD1 were divided in negative (IRS = 0) and positive (IRS ≥ 1).

### Statistical analysis

Statistical analyses were performed using SPSS 22.0 for Windows (IBM) and were previously described [23–25]. In short, in TMA1 logistic regression was used to evaluate associations between cytoplasmic/nuclear COMMD1 expression and patient characteristics. Survival analyses for disease-specific survival (DSS) and PFS were performed using the Cox proportional hazards model. None of the variables violated the proportional hazards assumption. In TMA1 DSS was defined as time from primary (debulking) surgery to death caused by ovarian cancer or last follow up alive. PFS was defined as time from primary surgery to progression/relapse of the disease, death, or last follow up. Median follow-up for the 126 HGSOC patients was 21 months. Only *P*-values ≤0.10 in the univariate analysis were included for multivariate analysis if applicable. For TMA2, we performed similar analysis as for TMA1.

*In vitro* data shown were derived from three independent experiments ± standard error of the means (SEM). Analyses were performed using GraphPad version 6.05 (GraphPad software). Student’s t-test was used to test the significance. For all experiments a *P*-value of <0.05 was considered as statistically significant.

## Results

### COMMD1 expression in human ovarian tumor samples

To assess the expression of COMMD1 in ovarian tumors, we first determined the specificity of the anti-COMMD1 antibody in HeLa and HEK293T cell lines, which were stable depleted for COMMD1 (Fig 1A and 1B) [1,19]. In contrast to the control cells, almost no staining of COMMD1 was detected in COMMD1-depleted cells (Fig 1A). These results certified the specificity of our immunohistochemical staining of COMMD1. Next, we stained COMMD1 in a panel of whole sections of ovarian cancer tissues (n = 21, Fig 1C), and observed COMMD1 expression in all samples. Interestingly, however, we detected a marked nuclear expression of COMMD1 in a subset of cancer tissue samples, in addition to cytoplasmic staining of COMMD1 (Fig 1C). Using semi-quantitative analysis of COMMD1 immunohistochemical stainings (Fig 1D), we indeed found consistently lower levels of nuclear COMMD1 in ‘nuclear COMMD1-negative’ tumor samples when compared to ‘nuclear COMMD1-positive’ tumor samples (Fig 1E).

### Increased nuclear expression of COMMD1 sensitizes ovarian cancer cells to cisplatin treatment

**Fig 2:**
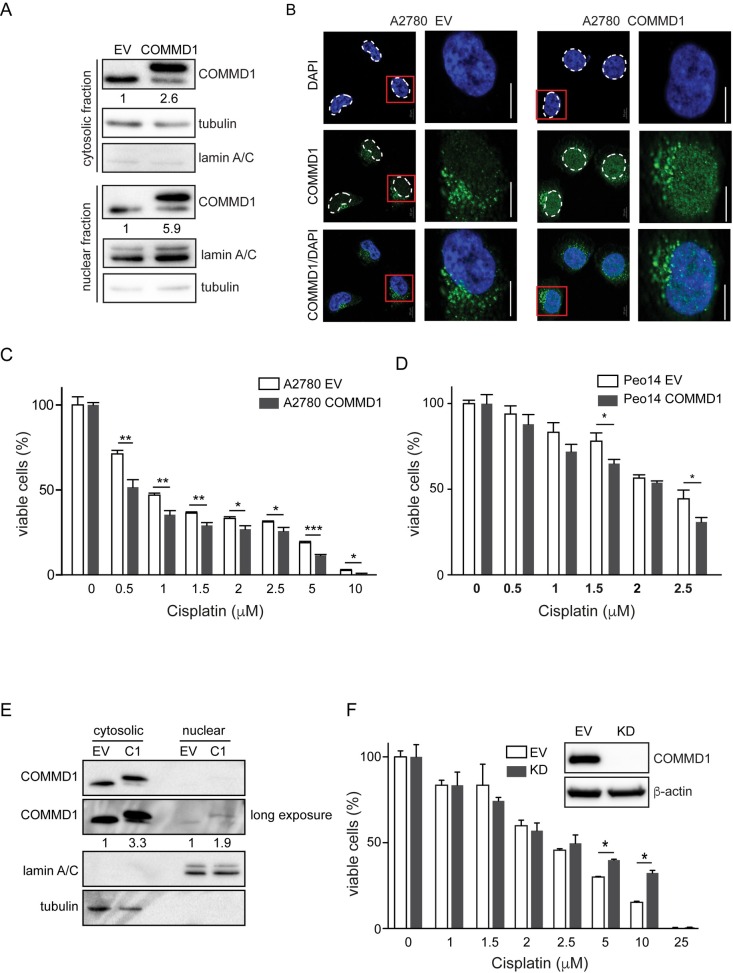
Increased nuclear COMMD1 expression in A2780 cells enhanced cisplatin sensitivity. **(A)** Subcellular localization of COMMD1 in A2780 EV and A2780-COMMD1 cells determined by immunoblotting. Intensity of individual bands for COMMD1 was quantified using ImageLab software. After correction for tubulin or lamin A/C expression the relative COMMD1 expression in A2780-COMMD1 cells was determined. **(B)** A2780 EV and A2780 COMMD1 cells were stained for COMMD1 (green), and DNA (blue) and imaged by fluorescent microscopy. The scale bar represents 20 μm. **(C)** A2780 EV and A2780 COMMD1 cells were plated in 96-well plates and treated with indicated concentrations of cisplatin. After 72 hours of treatment, cells were incubated with MTT for 3 hours and the viability of cells was determined by colorimetric measurement. Data are shown from three independent experiments. Statistical significance was calculated using the Student's t-test. *: *P*< 0.05, **: *P* <0.01, ***: *P* <0.001. **(D)** Overexpression of COMMD1 in Peo14 cells augments cisplatin sensitivity. Control cells (Peo14 EV) and Peo14 cells stably overexpressing COMMD1-Flag (Peo14 COMMD1) were plated in 96-well plates and treated with indicated concentrations of cisplatin. After 72 hours of treatment, cells were incubated with MTT for 3 hours and the viability of cells was determined by colorimetric measurement. Data are shown from three independent experiments. Statistical significance was calculated using the Student's t-test. *: *P*< 0.05. (**E**) Subcellular localization of COMMD1 in Peo14-EV and Peo14-COMMD1 cells determined by immunoblotting. Intensity of individual bands for COMMD1 was quantified using ImageLab software. **(F)** Silencing of COMMD1 results in decreased sensitivity of A2780 cells to cisplatin. Control cells (EV) and COMMD1 silenced A2780 cells (KD) were plated in 96-well plates and treated with indicated concentrations of cisplatin. After 72 hours of treatment, cells were incubated with MTT for 3 hours and the viability of cells was determined by colorimetric measurement. Data are shown from three independent experiments. Statistical significance was calculated using the Student's t-test. *: *P*< 0.05

The differential subcellular expression of COMMD1 in ovarian cancer, together with the notion that nuclear COMMD1 inhibits NF-κB and HIF activity and that COMMD1 might have a function in DNA damage response prompted us to evaluate whether nuclear COMMD1 affects the sensitivity of ovarian cancer cells to cisplatin [3,10]. To this end, we stably overexpressed FLAG-tagged COMMD1 in A2780 ovarian cancer cells. We previously showed that the FLAG tag does not interfere with COMMD1 function, as FLAG-tagged COMMD1 can rescue the lethal phenotype of *Commd1*^-/-^ mice [29]. Stable overexpression of COMMD1-FLAG in A2780 cells resulted in a 2.6-fold increase in cytosolic expression (Fig 2A) and 5.9-fold increase in nuclear COMMD1 levels compared to control-transfected cells (A2780-EV), in which COMMD1 is predominantly cytoplasmic (Fig 2A). Indirect immunofluorescence staining validated this increase in nuclear COMMD1 levels (Fig 2B). In the control cells we observed cytoplasmic vesicular localization of COMMD1 and a very weak nuclear staining, similar as previously reported for other cell lines [9–11,29]. However, in addition to a vesicular COMMD1 localization, A2780-COMMD1 cells also showed a significant increase in nuclear COMMD1 expression (Fig 2B). Since the expression of COMMD1 was predominantly increased in the nucleus of A2780-COMMD1 cells, we reasoned that these cells reflect the tumors with elevated nuclear COMMD1 expression (Fig 1C) and therefore represent an elegant model to study the role of aberrant COMMD1 expression on cisplatin sensitivity. Control A2780 cells (EV), and cells with high nuclear COMMD1 levels were treated with different concentrations of cisplatin for 72 hours after which the viability of cells was measured by MTT conversion. Stable nuclear COMMD1 expression resulted in decreased cell viability after treatment with 0.5–10 μM cisplatin (Fig 2C). Although to a lesser extend, similar results were seen in the ovarian cancer cell line Peo14 in which we stably increased COMMD1 expression (Fig 2D). This mild effect is likely due to the relative lower overall and nuclear increase in COMMD1 levels (Fig 2E) compared to A2780-COMMD1 cells (Fig 2A). Next, we determined the COMMD1 levels in A2780/Cp70 cells, an ovarian cancer cell line, which is 13-fold more resistant to cisplatin than A2780 cells [30]. A2780/Cp70 cells have reduced cytosolic and nuclear COMMD1 levels compared to A2780 cells (S1 Fig). To assess the effect of COMMD1 reduction in cisplatin sensitivity we decreased the COMMD1 levels by shRNA, which diminished cisplatin sensitivity of A2780 cells (Fig 2F). Taken together, these results show that nuclear levels of COMMD1 are associated with cisplatin sensitivity in ovarian cancer cells.

### Elevated nuclear COMMD1 expression ameliorates G_2_/M cell cycle checkpoint function upon cisplatin treatment

**Fig 3:**
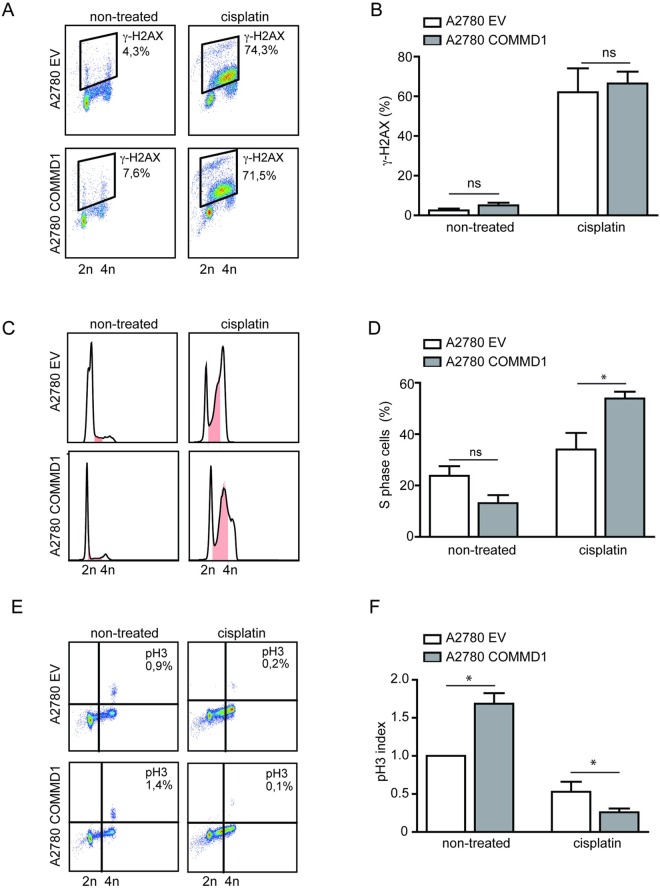
Elevated nuclear COMMD1 levels increase G_2_/M checkpoint after cisplatin treatment in A2780 cells. **(A)** A2780 cells treated with 2 μM cisplatin for 24 hours were harvested, fixed in 70% ethanol, stained with anti-γ-H2AX/Alexa-647 and analyzed by flow cytometry. Representative plots of at least 10,000 events are shown. **(B)** Percentages indicate average amounts (with SEM) of γ-H2AX-positive cells from three independent experiments. **(C)** A2780 cells treated with 2 μM cisplatin for 24 hours were harvested, fixed in 70% ethanol, stained with propidium iodide and cell cycle distribution was analyzed by fluorescence-activated cell sorting (FACS). Representative FACS profiles are shown. S-phase cell fraction is marked in pink. **(D)** Average percentages and SEM of S-phase cells from three independent experiments are presented. **(E)** A2780 cells treated with 2 μM cisplatin for 24 hours were harvested, fixed in 70% ethanol and stained with anti-p-H3/Alexa-488 and analyzed by flow cytometry. Representative plots of at least 10,000 events are shown. **(F)** Percentages indicate average amounts (with SEM) of p-H3 positive cells from three independent experiments. Significance was calculated using the Student's t-test (* represents *P*<0.05).

The fact that COMMD1 can interact with nuclear BRCT domain-containing proteins, led us to hypothesize that COMMD1 may play a role in the DNA damage response. Therefore, we assessed the effect of increased nuclear expression of COMMD1 on the level of DNA damage and cell cycle checkpoint behavior in response to cisplatin. The level of DNA breaks upon cisplatin treatment, as judged by gamma-H2AX foci formation, was not significantly different between the two cell lines (Fig 3A and 3B). Next, the contribution of nuclear COMMD1 on cell cycle checkpoint function was determined. First, proliferation rates of the control (EV) and A2780-COMMD1 cells were studied. As shown in Fig 3C, the proliferation rate was not affected by increased expression of nuclear COMMD1. However, as expected, cisplatin treatment (2 μM, 24 hours) resulted in accumulation of cells in S phase, which was more pronounced in A2780-COMMD1 cells compared to control cells (Fig 3C and 3D). Second, the percentage of cells that entered mitosis, as judged by the phosphorylation level of Histone-H3, was determined. After 24 hours of cisplatin treatment, a marked cell cycle arrest at G_2_ phase was observed in both cell lines (Fig 3E and 3F), but the percentage of mitotic cells was further decreased by elevated nuclear COMMD1 expression (Fig 3E and 3F). These results indicate that increased COMMD1 expression, along with elevated nuclear COMMD1, ameliorates G_2_/M checkpoint function in response to cisplatin treatment.

### Cisplatin-induced apoptosis is increased in ovarian cancer cells with elevated levels of nuclear COMMD1

**Fig 4:**
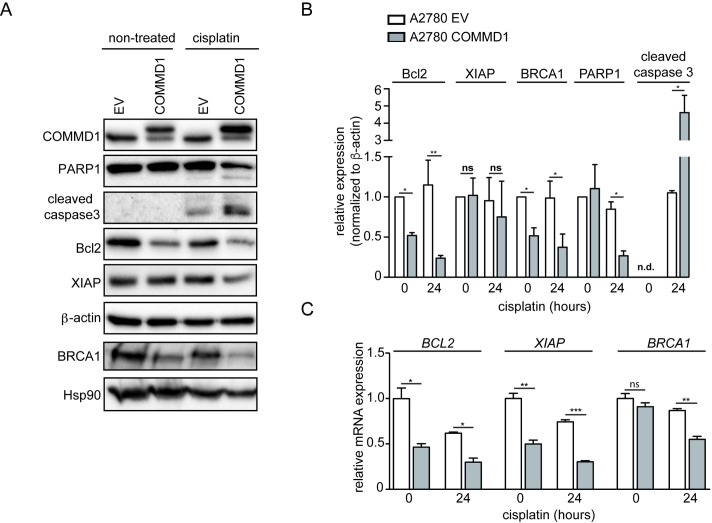
Increased nuclear expression of COMMD1 leads to elevated apoptosis and impaired gene expression in cisplatin treated A2780 cells. **(A)** Whole-cell lysates of untreated and 2 μM cisplatin (24 hours) treated A2780 EV and A2780 COMMD1 cells were analyzed by immunoblotting using the indicated antibodies. **(B)** Relative protein levels of Bcl2, XIAP, BRCA1 were quantified (n = 3) **(C)** mRNA levels of the indicated genes in non-treated and 2 μM cisplatin (24 hours) treated A2780 COMMD1 cells were determined by qRT-PCR and presented relative to A2780 EV control cells. GAPDH expression was used as an internal control. Average values are presented with SEM. Significance was calculated using the Student's t-test. *: *P*< 0.05, **: *P* <0.01, ***: *P* <0.001. **(D)** Representative immunostaining for both low and high cytoplasmic COMMD1 staining and negative or positive nuclear COMMD1 staining in HGSOC tumor samples. **(E)** Proposed model of how chemosensitivity for cisplatin is regulated by nuclear COMMD1 in A2780 EV and COMMD1 ovarian cancer cells.

Since apoptosis is the major process contributing to cell death induced by DNA damaging agents [31], we evaluated whether cells with higher nuclear COMMD1 expression levels more efficiently undergo apoptosis after cisplatin-mediated DNA damage. The apoptotic markers cleaved caspase-3 and cleaved PARP-1 were higher in A2780-COMMD1 compared to control cells after 24h of cisplatin treatment (Fig 4A). Next, we analyzed the expression of the anti-apoptotic proteins *BCL2* and *XIAP*. Independent of cisplatin treatment, we found that both protein and mRNA levels of *BCL2* were significantly lower (Fig 4A, 4B and 4C). Also, the mRNA levels of *XIAP*, a direct inhibitor of caspase-3, were reduced, independently of cisplatin treatment (Fig 4C). However, the protein levels of XIAP were not significantly decreased (Fig 4A and 4B). Furthermore, upon cisplatin incubation, a significant reduction in mRNA and protein levels of the DNA repair gene *BRCA1* was observed in A2780-COMMD1 cells (Fig 4A, 4B and 4C). Altogether, these results suggest that increased COMMD1 levels, including elevated COMMD1 levels, causes impaired expression of the indicated anti-apoptotic and DNA repair genes, which is accompanied by increased apoptosis after cisplatin treatment.

### COMMD1 expression in advanced stage HGSOC patients

**Fig 5:**
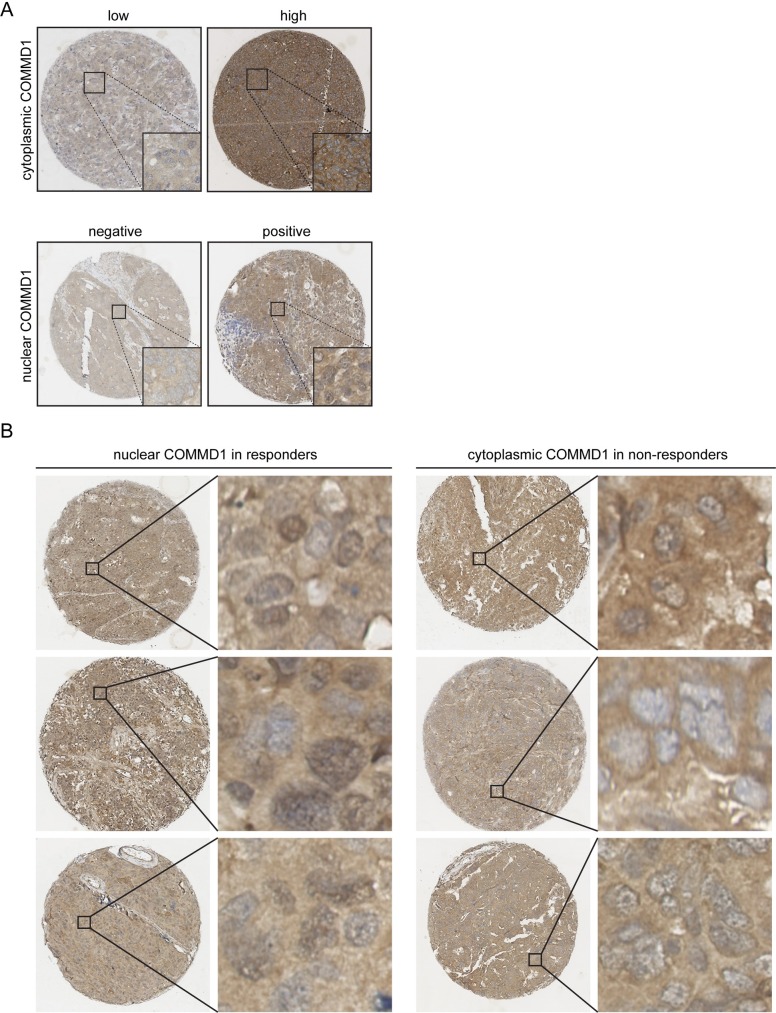
Examples of COMMD1 levels and localization in ovarian cancer samples. **(A)** Representative immunohistochemical stainings of cytoplasmic and nuclear COMMD1 are indicated. **(B)** Representative immunohistochemical stainings of cytoplasmic and nuclear COMMD1 of responders (n = 3) and non-responders (n = 3).

Given the nuclear expression of COMMD1 in a subgroup of ovarian cancer patients (Fig 1C) and increased cisplatin sensitivity of ovarian A2780 cancer cells with elevated nuclear COMMD1 levels (Fig 2C), we explored whether the expression and subcellular localization of COMMD1 in ovarian tumors correlated with patient outcome. To this end, we assessed the cytoplasmic and nuclear COMMD1 expression in chemo-naive tumor samples of 126 retrospectively selected advanced stage HGSOC patients (tissue microarray #1, TMA1). Of note, the tumor samples analyzed in Fig 1C are representing TMA1. In 94% of these tumor samples, COMMD1 expression was detected. Of those tumors with positive COMMD1 expression, cytoplasmic COMMD1 staining was readily detected (>75% of tumor cells stained positive) in all patients and only differed in intensity. We observed ‘high’ cytoplasmic expression in 43/120 (35.8%) patients, and nuclear expression of COMMD1 in 64/118 (54.2%) patients. Representative immunohistochemical stainings of cytoplasmic and nuclear COMMD1 are shown in Fig 5A. Patient characteristics are summarized S2 Table.

During the follow up period, 95/126 (75.4%) patients died because of ovarian cancer. Analysis of the entire patient group showed that high cytoplasmic or nuclear COMMD1 staining was not correlated with disease-specific survival (DSS) and progression-free survival (PFS) in univariate cox regression analysis (S3 Table). Residual disease of ≥2 cm after primary surgery was related with both DSS (HR = 1.755; *P* = 0.016) and PFS (HR 1.558; *P* = 0.045). We found no association (OR 0.257; *P* = 0.142) between ‘high’ cytoplasmic COMMD1 staining and chemotherapy response (Table 1). However, using subgroup analysis, nuclear COMMD1 expression was significantly less frequently observed in the non-responders group (OR = 0.167; *P* = 0.038) in this exploratory TMA1 (Table 1). Representative immunohistochemical stainings of cytoplasmic and nuclear COMMD1 of responders (n = 3) and non-responders (n = 3) (Fig 5B). This result suggests that nuclear COMMD1 is related with improved response to chemotherapy in a subgroup of advanced stage HGSOC patients.

**Table 1:** Response to therapy for TMA1: logistic regression analysis for advanced stage HGSOC patients with ≥2 cm residual disease after primary surgery, and receiving platinum-based chemotherapy and a PFS of <6 months (n = 14) versus PFS of >18 months (n = 16).

	OR	95% CI		*P*-value
		Lower	Upper	
Age (continuous)	0.969	0.915	1.026	0.286
Positive nuclear COMMD1	**0.167**	**0.031**	**0.904**	**0.038**
High cytoplasmic COMMD1	0.257	0.042	1.573	0.142

OR = odds ratio, CI = confidence interval

To confirm these observed associations for nuclear COMMD1 in the exploratory TMA1, we analyzed COMMD1 protein expression in a second patient cohort (TMA2, S4 Table). This patient cohort (n = 67) was prospectively collected as part of a clinical study (NTR1491). In 24 tumors, positive nuclear COMMD1 (35.8%) was seen, whereas in 33 tumors no nuclear COMMD1 staining was observed (49.3%). Using Cox regression analysis, no prognostic value of nuclear COMMD1 expression was found (DSS: HR = 1.396, *P* = 0.360; PFS: HR = 1.016, *P* = 0.960) (Table 2). To investigate whether nuclear COMMD1 in this patient cohort was associated with tumor response to chemotherapy as suggested from the exploratory TMA1 data, associations between nuclear COMMD1 were again analyzed in a subset of patients with distinct favorable or poor therapy responses. Of the 13 patients included in this analysis, 10 patients were considered as ‘responders’. Of these ‘responders’, 7 patients showed no nuclear COMMD1 expression, while 3 patients did. The 3 ‘non-responders’ showed nuclear COMMD1 expression. Due to the small amounts of patients and the absence of non-responders without nuclear COMMD1 expression in TMA2, no statistically meaningful analysis could be performed on this specific patient group.

**Table 2:** Disease-specific and progression-free survival analysis in relation to nuclear COMMD1 expression in tumors of patients with advanced stage HGSOC from TMA2.

**Disease-specific survival**				
	HR	95% CI		*P*-value
		Lower	Upper	
Age (continuous)	1.019	0.991	1.048	0.181
Residual disease				
None	Ref.			
Present	1.534	0.777	3.027	0.218
Unknown	3.503	1.122	10.939	0.031
No primary debulking	1.720	0.600	4.931	0.313
Nuclear COMMD1	1.396	0.684	2.850	0.360
**Progression-free survival**				
	HR	95% CI		*P*-value
		Lower	Upper	
Age (continuous)	1.015	0.988	1.044	0.280
Residual disease				
None	Ref.			
Present	1.974	1.021	3.816	0.043
Unknown	4.655	1.454	14.903	0.010
No primary debulking	2.378	0.927	6.103	0.072
Nuclear COMMD1	1.016	0.553	1.866	0.960

HR = hazard ratio, CI = confidence interval

## Discussion

We here found that nuclear COMMD1 is associated with the response to cisplatin in ovarian cancer cells *in vitro*, and might be related to response to cisplatin treatment in ovarian cancer patients. The observation that elevated expression of COMMD1 in the nucleus is related to responses to cisplatin is fascinating, since COMMD1 is predominantly expressed in the cytoplasm of most cell types [9–11]. Interestingly, its nuclear expression can be actively controlled via a nuclear export signal (NES) in an exportin 1 (CRM1)-dependent manner [10], indicating that under certain conditions the levels of nuclear COMMD1 can be tightly regulated.

The relation between subcellular COMMD1 localization and response of ovarian cancer patients to therapy and survival was not established previously. To our knowledge this is the first study that suggests a link between nuclear COMMD1 levels and therapy response. In our exploratory patient cohort (TMA1), we observed in a subgroup of advanced stage HGSOC patients an association between nuclear COMMD1 expression and response to therapy. Since the exploratory TMA1 was retrospectively collected, we wished to validate our findings in an independent patient cohort. We therefore analyzed tumor material of HGSOC patients from the DoCaCel study (NTR1491). Unfortunately, due to the limited number of available patient tumor specimens, we were unable to test our hypothesis in a second, study-based TMA.

**Fig 6:**
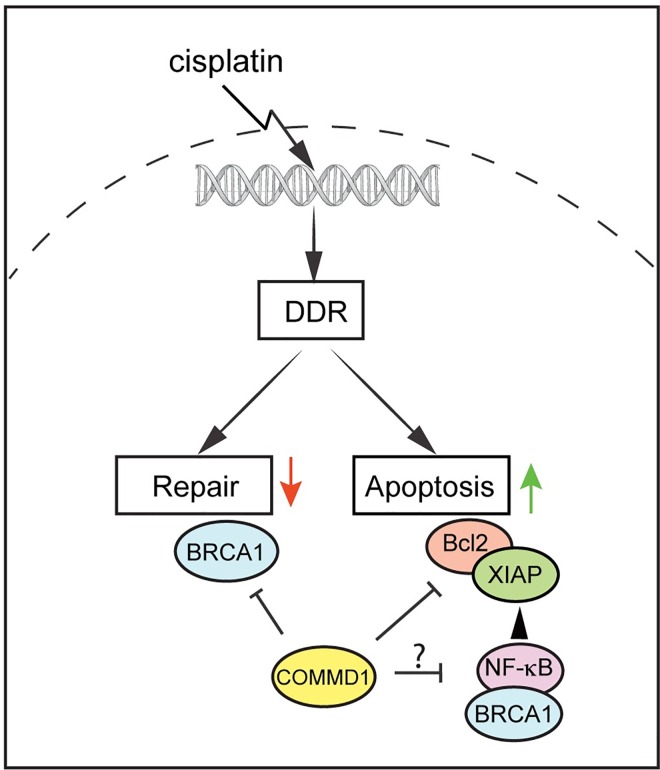
Model of how COMMD1 may influence responses to cisplatin.

Mechanistically, our *in vitro* data indicated that the improved cisplatin sensitivity by nuclear COMMD1 is possibly linked to reduced protein expression of the genes *BRCA1*, and *BCL2*. BRCA1 is a key component of the homologous recombination DNA repair pathway, and reduced expression or mutational inactivation of BRCA1 was reported to increase sensitivity to cisplatin [32]. In addition to its role in DNA repair, BRCA1 is also implicated in various other cellular functions, including transcription regulation, a processes which is also involved in the responsiveness of cells to cisplatin. In this context, BRCA1 was demonstrated to regulate the expression of the NF-κB anti-apoptotic target genes *BCL2* and *XIAP*, in a fashion dependent on the NF-κB subunit p50 [33]. Since COMMD1 inhibits NF-κB activity and can interact with BRCA1, it is tempting to speculate that nuclear COMMD1 represses the BRCA1-p50 mediated transcription of *BCL*2 and *XIAP* and thereby sensitizes ovarian cancer cells to cisplatin-induced apoptosis (model in Fig 6). This idea is further supported by the observation that nuclear COMMD1 is important to suppress transcription of NF-κB target genes [10,13–15].

It has been well established that COMMD1 also regulates the function of the copper transporters ATP7A and ATP7B [11,21,34–37], and both copper transporters have been implicated in cisplatin resistance [38–41]. The mechanism of ATP7A/B mediated cisplatin resistance remains unclear, but it has been postulated that these metal transporters might reduce the cytotoxic effect of cisplatin. However, we did not observe a significant difference in cisplatin-induced DNA damage as judged by gamma-H2AX formation, suggesting that increased COMMD1 expression does not affect the function of the copper-transporting ATPases in intracellular accumulation of cisplatin. Gamma-H2AX foci also reflects DNA breaks that are not repaired through BRCA1, and since gamma-H2AX remains associated with sites of DNA breaks even after repair, which could explain why decreased BRCA1 levels upon COMMD1 overexpression was not associated with altered gamma-H2AX foci numbers.

Remarkably, our results on the role of COMMD1 in cisplatin sensitivity are in contradiction with prior data that demonstrated that reduced levels of COMMD1 in a breast cancer cell line [3] and a hepatoma cell line [21] lead to increased cisplatin sensitivity. We were able to replicate the enhanced cisplatin sensitivity in the breast cancer cell line MDA-MB-231 upon COMMD1 silencing (S2 Fig), which would indicate that the effects of COMMD1 on cisplatin response and tumor behavior are cell-type specific. A cell-type specific role of COMMD1 in tumor behavior is corroborated by prior studies. In diffuse large B-cell lymphoma (DLBCL) high COMMD1 expression is correlated with poor prognosis [2] and is associated with aggressive forms of (DLBCL) [42]. Increased COMMD1 expression is also common in primary mediastinal B-cell lymphoma (PMBL) cells lines and in PMBL patients [43]. In contrast, in many other types of cancer (e.g. seminoma, ovarian, breast, and prostate cancer) a reduced expression of COMMD1 has been found. This decreased COMMD1 expression is correlated with cancer behavior and reduced survival rate [1,4,17,44].

Taken together, our data suggest that elevated levels of nuclear COMMD1 confer A2780 ovarian cancer cells to be more sensitive to platinum-based chemotherapy. In line with our *in vitro* data, we found that increased expression of COMMD1 in the nucleus of ovarian tumors is associated with improved response to cisplatin therapy. Better understanding of the mechanism that regulates nuclear expression of COMMD1 is required and might uncover novel therapeutic targets to improve cellular responses to platinum-based chemotherapeutics.

## Supporting Information

S1 Fig Reduced cytosolic and nuclear COMMD1 levels correlates with increased cisplatin resistance of A2780 ovarian cancer cells. Subcellular localization of COMMD1 in A2780 and A2780/Cp70 cells determined by immunoblotting.
(PDF)

S2 Fig Silencing of COMMD1 results in increased cisplatin sensitivity of breast cancer MDA-MB-231 cells. Control cells (EV) and MDA-MB-231 stably expressing shRNA against COMMD1 (KD) were plated in 96-well plates and treated with indicated concentrations of cisplatin. After 72 hours of treatment, cells were incubated with MTT for 3 hours and the viability of cells was determined by colorimetric measurement. Data are shown from three independent experiments. Statistical significance was calculated using the Student's t-test. *: *P*< 0.05.
(PDF)

S3 Fig Full scans of the western blots. (TIF)

S4 Fig Full scans of the western blots. (TIF)

S5 Fig Full scans of the western blots. (TIF)

S1 Table qPCR primer sequences. (DOCX)

S2 Table Patient and tumor characteristics of TMA1. (DOCX)

S3 Table Disease-specific and progression-free survival analysis of TMA1. (DOCX)

S4 Table Non-responders vs responders in TMA1. (DOCX)
