# Supplementary figures and images for: Nuclear COMMD1 Is Associated with Cisplatin Sensitivity in Ovarian Cancer
Source: PLoS One. 2016 Oct 27;11(10):e0165385. doi: 10.1371/journal.pone.0165385 (PMC5082896; doi:10.1371/journal.pone.0165385)

Figure S1

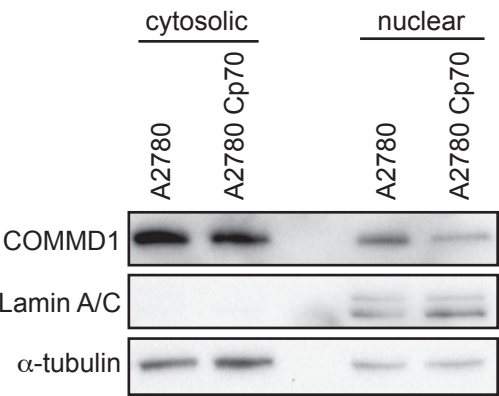

Supplement: S1 Fig — Subcellular localization of COMMD1 in A2780 and A2780/Cp70 cells determined by immunoblotting. (PDF) [file pone.0165385.s001.pdf]

Figure S2

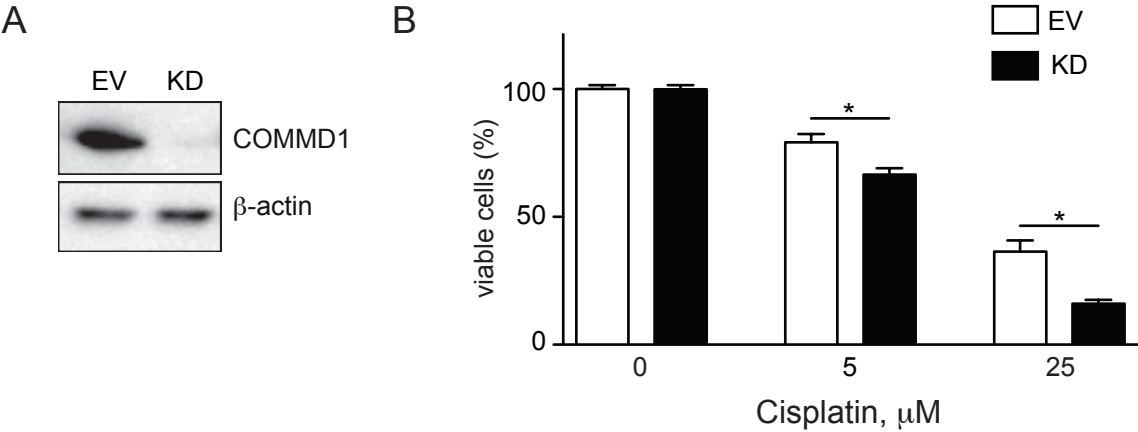

Supplement: S2 Fig — Control cells (EV) and MDA-MB-231 stably expressing shRNA against COMMD1 (KD) were plated in 96-well plates and treated with indicated concentrations of cisplatin. After 72 hours of treatment, cells were incubated with MTT for 3 hours and the viability of cells was determined by colorimetric measurement. Data are shown from three independent experiments. Statistical significance was calculated using the Student's t-test. *: P< 0.05. (PDF) [file pone.0165385.s002.pdf]

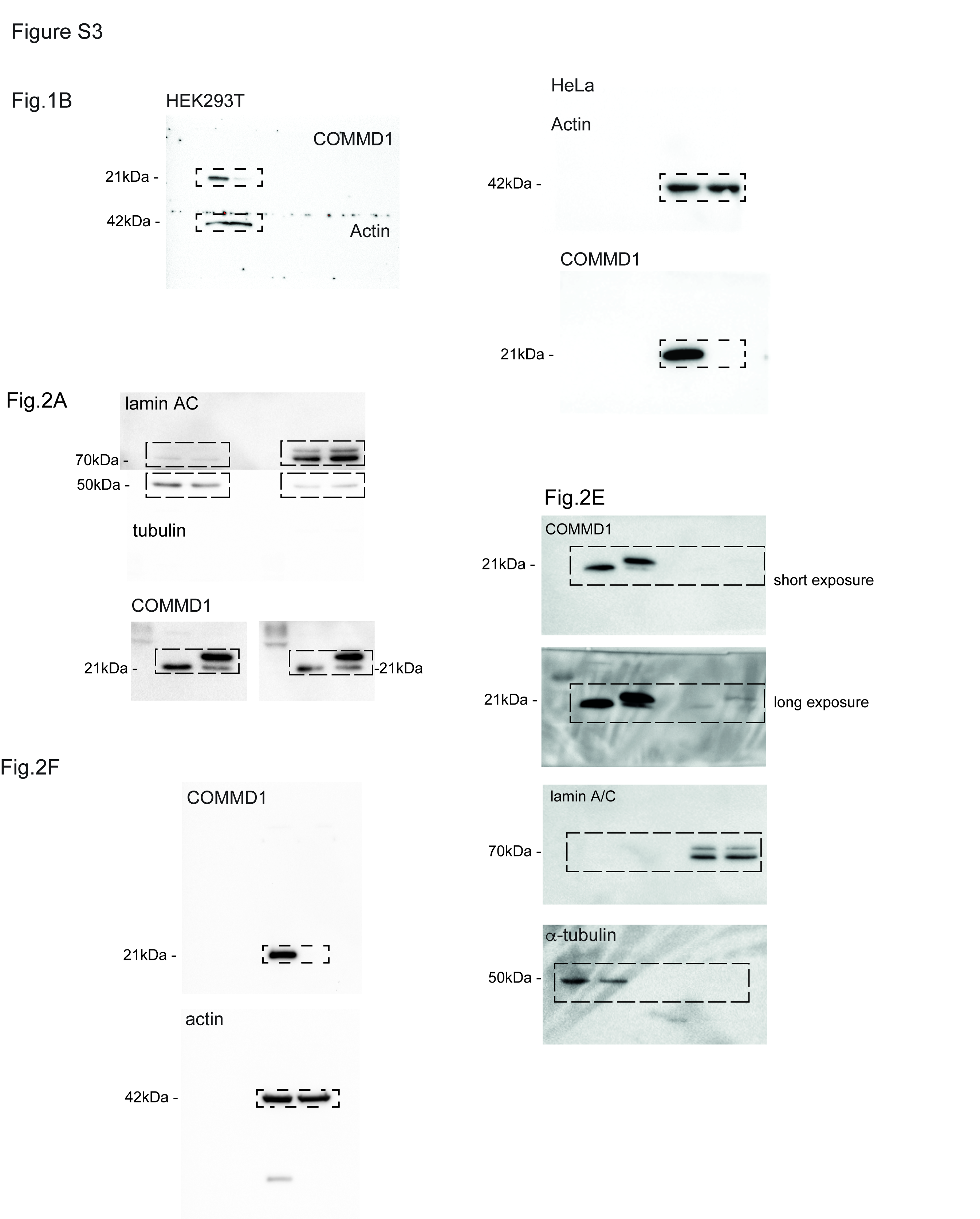

Supplement: S3 Fig — (TIF) [file pone.0165385.s003.tif]

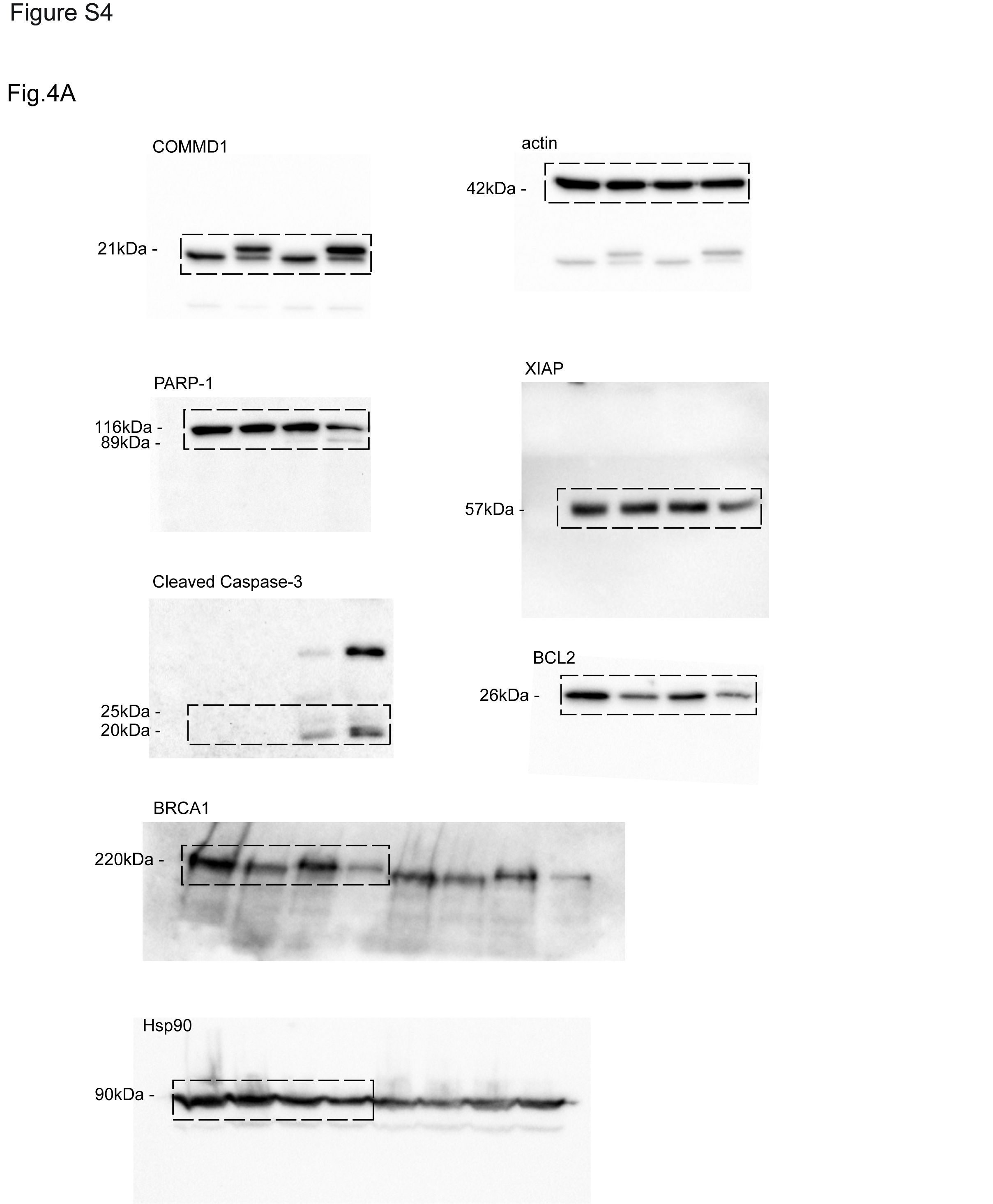

Supplement: S4 Fig — (TIF) [file pone.0165385.s004.tif]

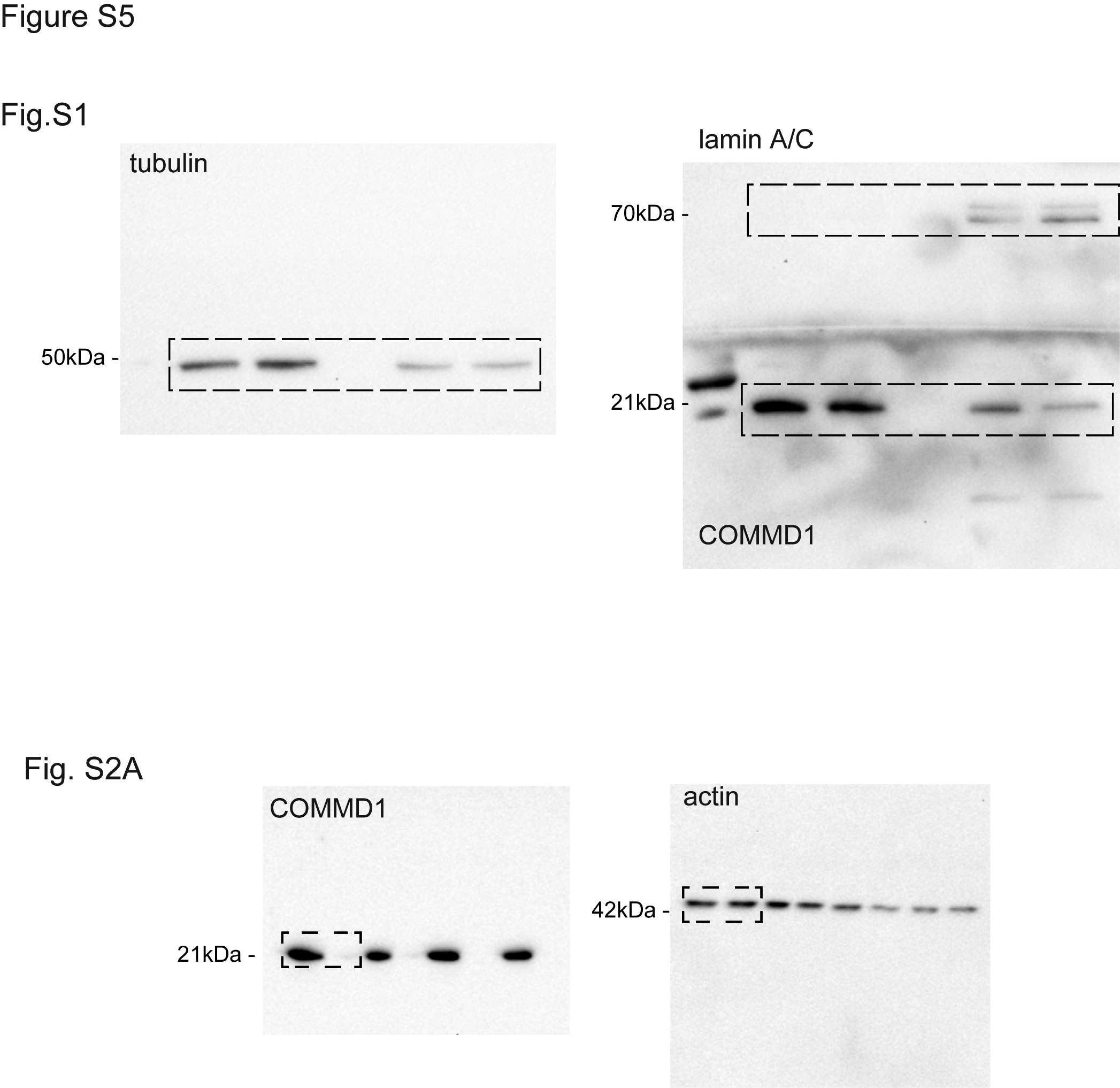

Supplement: S5 Fig — (TIF) [file pone.0165385.s005.tif]
