# Supplementary material for: Nuclear COMMD1 Is Associated with Cisplatin Sensitivity in Ovarian Cancer
Source: PLoS One. 2016 Oct 27;11(10):e0165385. doi: 10.1371/journal.pone.0165385 (PMC5082896; doi:10.1371/journal.pone.0165385)
Supplement: S1 Table — (DOCX) [file pone.0165385.s006.docx]

**Supplemental Table S1.** qPCR primer sequences.

| Gene | Forward 5’→3’ | Reverse 5’→3’ |
| --- | --- | --- |
| *BRCA1* | ATCATTCACCCTTGGCACA | GGAAGCCATTGTCCTCTGTC |
| *XIAP* | GCAAGAGCTCAAGGAGACCA | AAGGGTATTAGGATGGGAGTTCA |
| *BCL2* | GGATGACTGAGTACCTGAACC | GGCCGTACAGTTCCACAAAGG |
