# Supplementary material for: Nuclear COMMD1 Is Associated with Cisplatin Sensitivity in Ovarian Cancer
Source: PLoS One. 2016 Oct 27;11(10):e0165385. doi: 10.1371/journal.pone.0165385 (PMC5082896; doi:10.1371/journal.pone.0165385)
Supplement: S2 Table — (DOCX) [file pone.0165385.s007.docx]

| **Supplemental Table S2.** Patient and tumor characteristics of the 126 patients with advanced stage HGSOC for which cytoplasmic and nuclear COMMD1 expression was analyzed (TMA1). | | | |
| --- | --- | --- | --- |
|  |  | **n** | **(%)** |
| Age at diagnosis (years) |  |  |  |
| Median (range) | 61 (22-84) |  |  |
| Residual disease after primary surgery |  |  |  |
| <2 cm |  | 37 | (31.6) |
| ≥2 cm |  | 80 | (68.4) |
| System missing |  | 9 |  |
| Type of chemotherapy |  |  |  |
| No chemotherapy |  | 10 | (8.1) |
| Platinum containing |  | 38 | (30.6) |
| Platinum and taxane containing |  | 72 | (58.1) |
| Other regimen |  | 4 | (3.2) |
| System missing |  | 2 |  |
| Follow-up (months) |  |  |  |
| Median (range) | 21 (0-143) |  |  |
