# Supplementary material for: Nuclear COMMD1 Is Associated with Cisplatin Sensitivity in Ovarian Cancer
Source: PLoS One. 2016 Oct 27;11(10):e0165385. doi: 10.1371/journal.pone.0165385 (PMC5082896; doi:10.1371/journal.pone.0165385)
Supplement: S3 Table — (DOCX) [file pone.0165385.s008.docx]

| **Supplemental Table S3.** Disease-specific and progression-free survival analysis in relation to cytoplasmic and nuclear COMMD1 expression in tumors of patients with advanced stage HGSOC. | | | | |
| --- | --- | --- | --- | --- |
|  |  | | | |
| **Disease-specific survival** |  | | | |
|  | HR | 95% CI | | *P*-value |
|  |  | Lower | Upper |  |
| Age (continuous) | 1.007 | 0.987 | 1.027 | 0.517 |
| Residual disease ≥2 cm | **1.755** | **1.110** | **2.773** | **0.016** |
| Positive nuclear COMMD1 | 0.761 | 0.501 | 1.154 | 0.199 |
| High cytoplasmic COMMD1 | 0.901 | 0.586 | 1.384 | 0.633 |
|  |  | | | |
| **Progression-free survival** |  | | | |
|  | HR | 95% CI | | *P*-value |
|  |  | Lower | Upper |  |
| Age (continuous) | 0.995 | 0.976 | 1.015 | 0.626 |
| Residual disease ≥2 cm | **1.558** | **1.011** | **2.401** | **0.045** |
| Positive nuclear COMMD1 | 0.723 | 0.484 | 1.080 | 0.113 |
| High cytoplasmic COMMD1 | 0.967 | 0.644 | 1.452 | 0.872 |
| *HR = hazard ratio, CI = confidence interval* | | | | |
