# Supplementary material for: Nuclear COMMD1 Is Associated with Cisplatin Sensitivity in Ovarian Cancer
Source: PLoS One. 2016 Oct 27;11(10):e0165385. doi: 10.1371/journal.pone.0165385 (PMC5082896; doi:10.1371/journal.pone.0165385)
Supplement: S4 Table — (DOCX) [file pone.0165385.s009.docx]

| **Supplemental Table S4.** Patient and tumor characteristics of the 67 patients with advanced stage HGSOC for which nuclear COMMD1 expression was analyzed (TMA2). | | | |
| --- | --- | --- | --- |
|  |  | **N** | **(%)** |
| Age at diagnosis (years) |  |  |  |
| Median (range) | 62 (37-82) |  |  |
| Residual disease after primary surgery |  |  |  |
| None |  | 22 | (32.8) |
| Present |  | 33 | (49.3) |
| Unknown |  | 4 | (6.0) |
| No primary debulking |  | 8 | (11.9) |
| Follow-up (months) |  |  |  |
| Median (range) | 31 (0-85) |  |  |
